# CXCL5 and CXCL14, but not CXCL16 as potential biomarkers of colorectal cancer

**DOI:** 10.1038/s41598-023-45093-4

**Published:** 2023-10-17

**Authors:** Monika Zajkowska, Maciej Dulewicz, Agnieszka Kulczyńska-Przybik, Kamil Safiejko, Marcin Juchimiuk, Marzena Konopko, Leszek Kozłowski, Barbara Mroczko

**Affiliations:** 1 Department of Neurodegeneration Diagnostics, Medical University of Bialystok, 15-269, Bialystok, Poland; 2 Department of Oncological Surgery with Specialized Cancer Treatment Units, Maria Sklodowska-Curie Oncology Center, 15-027, Bialystok, Poland; 3 Department of Biochemical Diagnostics, Medical University of Bialystok, 15-269, Bialystok, Poland

**Keywords:** Chemokines, Biomarkers

## Abstract

Experts emphasize that colorectal cancer (CRC) incidence and mortality are increasing. That is why its early detection is of the utmost importance. Patients with cancer diagnosed in earlier stages have a better prognosis and a chance for faster implementation of treatment. Consequently, it is vital to search for new parameters that could be useful in its diagnosis. Therefore, we evaluated the usefulness of CXCL5, CXCL14 and CXCL16 in serum of 115 participants (75 CRC patients and 40 healthy volunteers). Concentrations of all parameters were measured using Luminex. CRP (C-reactive protein) levels were determined by immunoturbidimetry, while levels of classical tumor markers were measured using CMIA (Chemiluminescence Microparticle Immunoassay). Concentrations of CXCL5 were statistically higher in the CRC group when compared to healthy controls. The diagnostic sensitivity, specificity, positive and negative predictive value, and area under the ROC curve (AUC) of CXCL5 and CXCL14 were higher than those of CA 19–9. Obtained results suggest the usefulness of CXCL5 and CXCL16 in the determination of distant metastases and differentiation between TNM (Tumor-Node-Metastasis) stages, as well as the usefulness of CXCL14 and CRP combination in CRC detection (primary or recurrence). However, further studies concerning their role in CRC progression are crucial to confirm and explain their diagnostic utility and clinical application as biomarkers.

## Introduction

Annually, approximately 2,000,000 new cases of intestinal malignant neoplasm (ICD-10 Classification: C18-C20) are diagnosed. Analyzing the incidence structure, it can be concluded that colorectal cancer is the third most common malignant neoplasm afflicted by women and the second most common among men. Experts emphasize that the incidence and mortality of colorectal cancer in both sexes increases year-to-year. Early detection of colorectal neoplasms and rectal cancer is of utmost importance for the effectiveness of the therapy. Patients with cancers diagnosed in earlier stages have an enhanced prognosis and a chance for faster implementation of cancer treatment. In this context, it is worth remembering about the possibility of taking advantage of screening for colorectal cancer^1^.

Most cases of colorectal cancer are diagnosed in patients over 50 years of age, and very rare in patients under the age of 40. The disease usually progresses slowly and the symptoms of colorectal cancer are partially dependent on the location of the malignant processes. In the early stages of the disease, the symptoms of colorectal cancer are usually non-specific and have the form of abdominal pain and flatulence, which may suggest less serious problems with the gallbladder or peptic ulcer disease. Most patients with colorectal cancer do not report significant symptoms, or they are slight or nonspecific (matching many different digestive system diseases). In many cases, the cancer process is asymptomatic. Mentioned symptoms, if any, are often underestimated and not equated with threats such as colorectal cancer. Stool abnormalities are most often attributed to stress and poor diet. In addition to neoplastic changes, they can also be caused by inflammation in the large intestine, food poisoning or infections with intestinal parasites^2–4^.

The most commonly used techniques in CRC detection are colonoscopy and sigmoidoscopy. In some cases, imaging diagnostics, computed tomographic colonography or magnetic resonance method, are used. Even though significant improvement has been made in this area in recent years, in the case of small lesions, these procedures might be ineffective. Alternative diagnostic tools, useful in the discovery of colorectal cancer are tumor markers, which are synthesized mostly by tumor cells. Tumor markers have a particular utility not only in detecting malignancies and determining tumor advancement, but also in monitoring of treatment and early detection of recurrence^3,4^. The examples of tumor markers engaged in the identification of CRC are CEA (carcinoembryonic antigen) and CA 19–9 (cancer antigen 19–9). Regrettably, the diagnostic usefulness of these biomarkers is relatively low as they are not specific to the CRC itself^5^. Taking into account the above evidence, there is a critical need to find novel biomarkers, the use of which will allow for early recognition (primary or recurrence) of emerging cancer earlier than it was previously possible.

Increasing evidence suggests that small inflammatory cytokines (8–12 kDa) known as chemokines are key regulators of angiogenesis, including pathological angiogenesis. In chemical terms, chemokines are peptides composed of 70–130 amino acids^6^, structurally and functionally similar to growth factors. They are characterized by 20–50% sequence homology between molecules, which is reflected in their structural similarities. According to the nomenclature, the names of individual types of these compounds are created by adding the letter L (ligand) together with a sequential number. Most of the compounds in their structure contain four characteristic cysteine residues which, by forming disulfide bridges, determine their three-dimensional structure. Chemokines, on the basis of this structure, have been classified into 4 groups: CC, CXC, CX3C and X chemokines^7,8^.

The present state of knowledge allows us to suspect that all these proteins play a significant role in cancer advancement^9^. In cancer development and metastasis, these chemokines exert a complex outcome on angiogenesis, tumor cell proliferation and apoptosis regulation, facilitating tumor cell metastasis in an organ specific manner^10^. It is postulated that the CC and CXC chemokines could be the most active in the regulation of angiogenesis^11,12^. Initially, in tumor structure, blood vessels are not observed. However, in the course of cancer growth, when the tumor microenvironment begins to lack oxygen and nutrients, cancer cells begin to secrete substances that initiate angiogenesis processes. It has been shown that CXC-type chemokines secreted into the tumor microenvironment by tumor-associated macrophages and cancer cells are largely responsible for the regulation of angiogenesis. CXC chemokines, in addition to their pro-angiogenic properties, stimulate tumor growth and promote the formation of metastases, which takes place with the use of vessels formed in the processes of angiogenesis^8^. Early detection is crucial for patient prognosis and subsequent overall survival in the case of colorectal cancer, which is complicated by resistance to drugs and heterogeneity, especially in advanced stages where the process of angiogenesis is still active. The intricacy of the tumor microenvironment is largely attributed to the chemokines that are generated by the various cancer cells. Chemokine receptors on the surface of monocytes can interact with chemokines secreted by other cells. The monocytes can develop into macrophages and invade the tumor microenvironment after adhering. One of the causes of the heterogeneity in the CRC is the tumor associated macrophages (TAMs) that infiltrate the tumor^13^. That is why the aim of our study was an effort to elucidate and evaluate the utility of selected CXC-chemokines determination (CXCL5, CXCL14 and CXCL16) in patients with colorectal cancer compared to healthy control. We have also compared the obtained results to CA 19–9, CEA, and inflammatory parameter such as C-reactive protein (CRP).

## Materials and methods

### Patients

The study included 75 patients diagnosed by the oncology group for colorectal cancer (CRC) (Table 1). All patients were treated in the Maria Sklodowska-Curie Oncology Center, Department of Oncological Surgery with Specialized Cancer Treatment Units, Bialystok, Poland. Tumor classification and staging were conducted in accordance with the UICC-TNM (International Union Against Cancer Tumor-Node-Metastasis) classification.

**Table 1:** Characteristics of colorectal cancer and healthy patients groups.

Study group		No. of patients
Colorectal cancer		75 (100%)
	Gender	
	Female	26 (35%)
	Male	49 (65%)
	Type	
	Colon cancer	25 (33%)
	Rectal cancer	41 (55%)
	Sigmoid cancer	9 (12%)
	TNM stage	
	0	1 (1%)
	I	15 (20%)
	II	13 (17%)
	III	25 (34%)
	IV	21 (28%)
	Depth of tumor invasion	
	In situ	1 (1%)
	T1	2 (3%)
	T2	19 (25%)
	T3	41 (55%)
	T4	12 (16%)
	Nodal involvement	
	N0	34 (45%)
	N1	25 (34%)
	N2	16 (21%)
	Distant metastasis	
	M0	54 (72%)
	M1	21 (28%)
	Age	33–89
Control group		40 (100%)
	Gender	
	Female	12 (30%)
	Male	28 (70%)
	Age	34–80

Colorectal cancer histopathology was based on the microscopic examination of tissue samples. Moreover, all patients were grouped according to tumor stage (TNM), depth of tumor invasion (T factor), presence of lymph node (N factor) and distant metastases (M factor) as well as the histological grade (G factor) of the tumor. The pretreatment staging procedures included physical and blood examinations, CT scans (computed tomography) and in case of patients with rectal cancer – MRI (magnetic resonance imaging) of the small pelvis. Additionally, all patients were assessed according to the ECOG score (Eastern Cooperative Oncology Group). The control group included 40 healthy volunteers. For each patient qualified for the control group, the exclusion criteria such as: obesity, active infections and symptoms of an infection, respiratory diseases, digestive tract diseases or systemic diseases were applied. In both groups, patients with BMI (Body Mass Index) > 35 were not included.

### Biochemical analyses

The biochemical analysis of the tested parameters was performed as described previously^14,15^. Each participant’s blood was drawn into a tube with a clot activator (S-Monovette, SARSTEDT, Germany), centrifuged to separate the serum, and then kept at -80^0^C until being analyzed. We have used the Luminex Human Discovery Assay plates (R&D Systems, Abingdon, UK) and Luminex 200 analyzer to measure tested proteins. For each standard, control, and sample, duplicate samples were evaluated. The manufacturer’s instructions were followed when using the immunoturbidimetric method (Abbott, Chicago, IL, USA) to analyze CRP concentration and the chemiluminescent microparticle immunoassay (CMIA) to detect the levels of classical tumor markers in the serum.

### Statistical analysis

Statistical analysis was performed by RStudio and Statistica 13.0 as described previously^14,15^. The results of the initial statistical analysis, which employed the Shapiro–Wilk test, showed that neither the levels of the tumor markers nor the examined parameters followed a normal distribution. As a result, statistical comparisons between the groups were made using the *U*-Mann Whitney test, the Kruskal–Wallis test, and a post-hoc Dwass-Steele-Crichlow-Flinger test for multivariate analysis of diverse data. Using the cut-off values determined by the Youden’s index, the diagnostic sensitivity (SE), specificity (SP), and the predictive values of positive and negative test results (PPV and NPV, respectively) were calculated. The cut-off points for each of the tested parameters were designated as follows: CXCL5 – 953.96 pg/mL, CXCL14 – 650.92 pg/mL, CXCL16 – 926.01 pg/mL, CA 19–9 – 5.30 U/mL, CEA – 1.70 ng/mL, CRP – 2.50 mg/L. In order to assess the diagnostic accuracy, we also defined the receiver-operating characteristics (ROC) curve for each parameter and ran a Spearman's rank correlation test. Comparisons with a p-value of 0.05 or above were considered statistically significant.

### Ethics committee approval

The study was conducted according to the guidelines of the Declaration of Helsinki, and approved by the Ethics Committee of Medical University of Bialystok (R-I-002/564/2019; 28.11.2019).

### Informed consent

Informed consent was obtained from all participants involved in the study.

## Results

### CXC chemokines

Concentration of CXCL5, CXCL14, CXCL16, CA 19–9, CEA and CRP in sera of CRC patients and healthy patients (control group) were presented in Table 2. The non-parametric test (*U* Mann–Whitney) which compared the levels obtained in the above mentioned groupsrevealed that the concentration of CXCL5, CEA and CRP in the total CRC group were statistically higher, and CXCL14 significantly lower when compared to healthy controls (in all cases p < 0.05).

**Table 2:** Serum levels of tested parameters in cancer and control groups.

Parameter		Colorectal cancer	Control group	p*
CXCL5 [pg/mL]	Me	1299.8	894.2	**0.01**
	Min–Max	185.2–7500.0	204.2–2634.5	
CXCL14 [pg/mL]	Me	595.6	671.5	**0.03**
	Min–Max	312.3–8654.0	338.0–1968.8	
CXCL16 [pg/mL]	Me	927.2	910.2	0.67
	Min–Max	288.9–2876.0	540.4–1307.1	
CA 19–9 [U/mL]	Me	5.3	5.4	0.82
	Min–Max	2.1–8199.9	2.1–33.3	
CEA [ng/mL]	Me	3.9	1.0	** < 0.001**
	Min–Max	0.5–3688.0	0.5–15.6	
CRP [mg/L]	Me	6.0	1.4	** < 0.001**
	Min–Max	1.0–248.5	0.2–5.8	

*U Mann–Whitney test.

Significant values are in bold.

Furthermore, we performed a more exhaustive investigation with the use of two different tests used in statistics (Kruskal–Wallis and Dwass–Steel–Critchlow–Fligner). That is why we have divided the group in which colorectal cancer patients were included into four subgroups with the use of TNM grading into I–IV advancement groups. After Kruskal–Wallis analysis, we obtained significant results for CXCL5, CXCL14 and comparative parameters (Table 3). Taking the acquired results, it can be suggested that the CXCL5, CEA, and CA 19–9 concentration rises with the development of tumor. Interestingly, the CXCL14 analysis reveals that there is a significant difference between the control group and I stage of CRC, which in our opinion is a very important observation as it may serve as a potential marker of early neoplastic changes.

**Table 3:** Kruskal–Wallis and Dwass-Steel-Crithlow-Fligner tests analysis results.

Parameter		CXCL5	CXCL14	CXCL16	CA 19–9	CEA	CRP
Kruskal–Wallis *p*-value		** < 0.001**	**0.04**	0.07	**0.003**	** < 0.001**	** < 0.001**
Dwass-Steel-Crithlow-Fligner *p*-value	Control vs. I	0.98	**0.02**	0.42	0.19	0.34	** < 0.001**
	Control vs. II	0.85	0.99	0.87	0.83	0.08	** < 0.001**
	Control vs. III	0.21	0.78	0.88	0.50	**0.002**	** < 0.001**
	Control vs. IV	** < 0.001**	0.80	0.07	0.25	** < 0.001**	** < 0.001**
	I vs. II	0.82	0.12	0.74	0.98	1.00	0.99
	I vs. III	0.23	0.18	0.19	**0.033**	0.77	1.00
	I vs. IV	**0.004**	0.61	0.07	**0.013**	** < 0.001**	0.99
	II vs. III	0.93	0.98	0.63	0.17	0.68	0.95
	II vs. IV	**0.048**	0.97	0.08	0.15	** < 0.001**	0.99
	III vs. IV	0.39	1.00	0.35	0.99	**0.005**	0.97

Significant values are in bold.

As the number of cases in stage I and II of TNM classification was lower than 20, which may have an impact on the correctness of the acquired outcomes, we wanted to check their exactness using another test. We separated all colorectal cancer patients into two groups: less-advanced (TNM I + II) and advanced tumors (TNM III + IV). Furthermore, we separated the group of advanced tumors into single TNM’s (III and IV) due to the satisfactory number of patients in each stage to perform an accurate investigation in those groups and in comparison to healthy volunteers (control). Remarkably, we have observed significant differences between healthy patients and IV stage CRC in the case of CXCL5 and CXCL16, which may suggest its involvement in distant metastasis processes and what is of utmost importance, significant differences between all CRC stages (same as CA 19–9). In the case of CXCL14, we have observed differences between control and less-advanced stages (I and II) of CRC, which, as previously mentioned, is an extremely important result considering the fact, that there are no widely available, minimally-invasive methods that would be useful in case of asymptomatic patients (Table 4).

**Table 4:** U Mann–Whitney test analysis results between control group and TNM subgroups.

Parameter		CXCL5	CXCL14	CXCL16	CA 19–9	CEA	CRP
U Mann–Whitney test *p*-value	Control vs. I + II	0.99	**0.05**	0.13	**0.05**	**0.04**	** < 0.001**
	Control vs. III + IV	** < 0.001**	0.30	0.11	0.10	** < 0.001**	** < 0.001**
	Control vs. III	0.11	0.43	0.67	0.30	** < 0.001**	** < 0.001**
	Control vs. IV	** < 0.001**	0.45	**0.03**	0.14	** < 0.001**	** < 0.001**
	I + II vs. III + IV	** < 0.001**	0.31	** < 0.001**	** < 0.001**	** < 0.001**	1.00
	I + II vs. III	**0.05**	0.27	**0.02**	** < 0.001**	0.10	0.70
	I + II vs. IV	** < 0.001**	0.56	**0.001**	** < 0.001**	** < 0.001**	0.67

Significant values are in bold.

Table 5 shows the parameters of diagnostic utility of all tested parameters, except for CXCL16, which was the only newly tested parameter that did not show statistical significance.

**Table 5:** Diagnostic criteria of tested parameters in patients with colorectal cancer.

Tested parameters	Diagnostic criteria	Colorectal cancer
CXCL5	SE	68%
	SP	58%
	PPV	75%
	NPV	49%
	AUC	0.65
CXCL14	SE	67%
	SP	58%
	PPV	75%
	NPV	48%
	AUC	0.62
CA 19–9	SE	51%
	SP	48%
	PPV	64%
	NPV	34%
	AUC	0.51
CEA	SE	75%
	SP	70%
	PPV	82%
	NPV	60%
	AUC	0.79
CRP	SE	73%
	SP	78%
	PPV	86%
	NPV	61%
	AUC	0.84

We showed that the highest sensitivity (SE) from all parameters revealed CXCL5 and CXCL14 (68 and 67%, respectively). The detected value is slightly lower than SE of CEA (75%) and CRP (73%), but much higher than SE of CA 19–9 (51%). What is more, the specificity (SP) of CXCL5 and CXCL14 showed high value (both 58%), but was lower than the SP of CRP (78%) and CEA (70%), nevertheless higher than CA 19–9 (48%). Positive predictive value (PPV) was high for CXCL5 and CXCL14 (both 75%). The negative predictive value (NPV) was calculated at 49% for CXCL5 and 48% for CXCL14. All these values were slightly lower than the PPV and NPV of CEA and CRP. Additionally, all utility values of the newly tested parameters (CXCL5, CXCL14) were greater than those obtained for CA 19–9, which suggests their higher utility than this routine marker for colorectal cancer patients.

**Figure 1:**
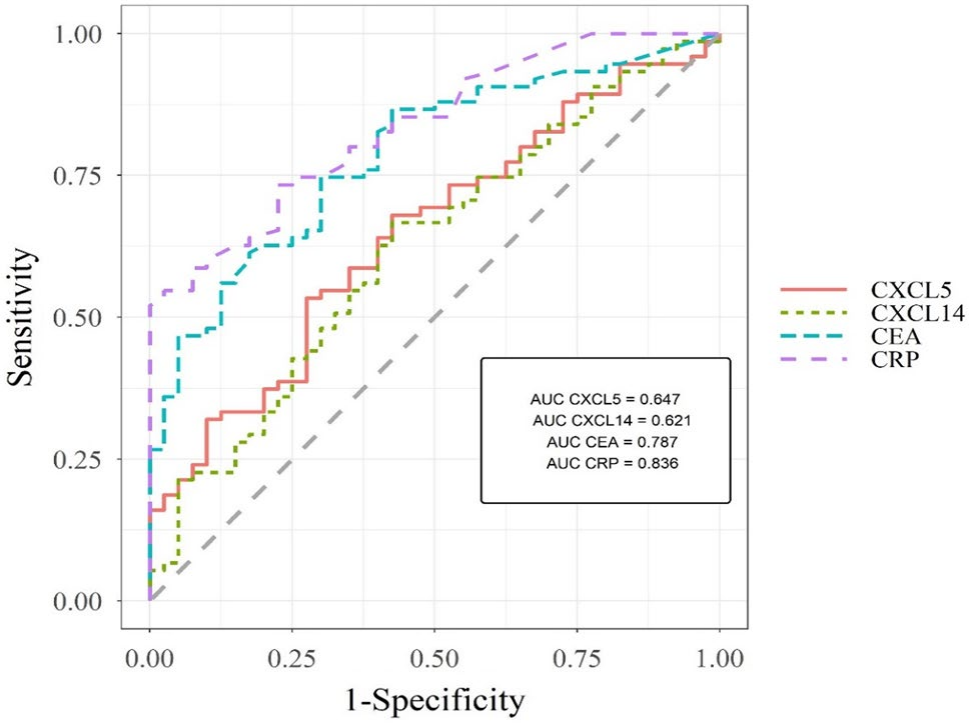
Receiver operating characteristics for all significant ROC analysis results (p < 0.05 in all cases). *AUC* area under ROC curve, *CXCL* C-X-C motif chemokine ligand, *CEA* carcinoembryonic antigen, *CRP* C-reactive protein.

We observed that the AUC of CXCL5 (0.65) in the total group of CRC was highest from all obtained results, but lower than the area under the ROC curve obtained for CEA and C-reactive protein. Nevertheless, in the case of both assessed CXC chemokines, AUC was higher than the AUC of CA 19–9 (Fig. 1).

We have also tested the Spearman’s rank correlation coefficient to indicate the strength and direction of monotonic association between variables. All results are presented in Table 6.

**Table 6:** Spearman’s rank correlation coefficient for tested variables.

Tested variables	CXCL5	CXCL14	CXCL16	CA 19–9	CEA	CRP	Age
CXCL14	0.06 *p* = 0.60	–					
CXCL16	**0.32 *p* = 0.005**	0.17 *p* = 0.16	–				
CA 19–9	**0.24 *p* = 0.04**	0.09 *p* = 0.44	0.22 *p* = 0.06	–			
CEA	**0.46 *p* < 0.001**	0.05 *p* = 0.68	**0.43 *p* < 0.001**	**0.51 *p* < 0.001**	–		
CRP	**0.25 *p* = 0.03**	0.06 *p* = 0.61	**0.47 *p* < 0.001**	− 0.01 *p* = 0.91	0.17 *p* = 0.16	–	
Age	0.04 *p* = 0.76	− 0.13 *p* = 0.27	− 0.01 *p* = 0.92	0.22 *p* = 0.06	**0.36 *p* < 0.001**	− 0.07 *p* = 0.55	–
TNM stage	**0.47 *p* < 0.001**	0.15 *p* = 0.20	**0.42 *p* < 0.001**	**0.43 *p* < 0.001**	**0.57 *p* < 0.001**	0.05 *p* = 0.67	**0.34 *p* = 0.003**

Significant values are in bold.

We observed a moderate positive correlation for CXCL5, CXCL16, as well as CEA and CA 19–9 with tumor TNM stage. This may indorse (as observed previously) that the growing levels of those factors are associated with the number of tumor cells. Moderate, positive correlation was also detected between CXCL5, CXCL16 with CEA concentration, CXCL16 with CRP levels, and both markers (CEA and CA 19–9). The rest of witnessed significant correlations revealed weak strength (coefficient < 0.40).

### CXC and CC chemokines combination

In order to continue the research on the usefulness of different chemokine groups in the diagnosis of colorectal cancer, we decided to use the previous assays^15^ in a combined analysis with the currently obtained concentrations. The obtained results turned out to be very interesting, which, in our opinion, significantly influences the validity of the published work. Significant combinations of all tested chemokine analyses were presented in Table 7 (all p < 0.001).

**Table 7:** Diagnostic criteria of chemokine combinations in patients with colorectal cancer.

Tested parameters	Diagnostic criteria	Colorectal cancer
CCL2/CRP ratio	SE	55%
	SP	85%
	ACC	75%
	AUC	0.80
CXCL14 + CRP	SE	83%
	SP	75%
	ACC	80%
	AUC	0.88
CEA + CRP	SE	79%
	SP	75%
	ACC	77%
	AUC	0.88

The obtained results suggest that the simultaneous analysis of the two parameters determination significantly influences the area under the ROC curve (AUC), which confirms the usefulness of the parameters tested. The performed comparative analysis for the routine marker (CEA) and CRP (Table 7) additionally confirms the usefulness of CXCL14 and suggests the need to continue research on this parameter and its use in the diagnosis of CRC, as the values of the obtained diagnostic criteria indicate greater usefulness of simultaneous CXCL14 and CRP determinations. In order to complete the statistical analysis, we also checked whether there were any correlations between the previously and currently studied chemokines. As a result of this analysis, we found a moderate positive correlation between CCL4 and CXCL5 (r = 0.46; p < 0.001) as well as CCL4 and CXCL16 (r = 0.54; p < 0.001).

## Discussion

The search for new biomarkers that could prove their usefulness in cancer screening remains a serious global problem. Early diagnosis, especially concerning the non-invasive lesions, remains unattainable. In the case of colorectal cancer, but also other neoplasms, researchers are constantly searching for biomarkers which would indicate the presence of neoplastic or even pre-neoplastic changes at the earliest possible stage and would replace or supplement the currently used imaging or histopathological tests. Similar assumptions apply when detecting disease recurrence after treatment^4^. There are few studies on the usefulness of selected CXC-chemokines in the course of colorectal cancer, which prompted us to carry out the above analysis. As CXC chemokines have been recognized as proteins with pro-angiogenic properties, we hope that changes in their concentrations will allow their identification at an early stage of cancer processes, even before the effective expansion of blood vessels within the tumor. The process of angiogenesis as one of the most important processes in the development of cancer lesions, involved in the formation of distant metastases, is an important aspect of this disease^8^.

We indicated that the serum concentration of CXCL5 was statistically higher in the group of colorectal cancer patients when compared to healthy controls (p = 0.01). Similar results were obtained in the work of Kawamura et al.^16^. These authors also revealed that preoperative serum levels of CXCL5 were significantly elevated in patients with CRC compared with healthy volunteers. Interestingly, the researchers pointed out that high serum CXCL5 was also associated with female sex, liver metastasis and poor overall survival. In addition, they have also measured the CXCL5 level in CRC cell lines, which confirmed previously mentioned results. These authors concluded, that CXCL5 preoperative serum level could serve as a novel predictive marker for prognosis determination of CRC which fully corresponds with our results. Different researchers in the work of Yildirim et al.^17^ pointed out that serum CXCL5 and CEA levels were significantly higher in the CRC group. In addition, these authors also performed immunohistochemical tests which revealed a high number of cases which were stained positive in the CRC group. Although the studies by Yilidirim et al.^17^ were carried out on a small study group, in which additionally a group of patients with benign lesions was specified, neither the CXCL5 concentration nor tissue expression differed when compared to the study group. This may indicate low usefulness of CXCL5 in the detection of benign lesions, but on the other hand, it can be a useful parameter when detecting tumor initiation. These assumptions require further analysis and confirmation using a much larger study group. In contrast, we also found work by Dimberg et al.^18^, in the course of which the researchers showed that the concentration of the CXCL5 protein in homogenates of neoplastic tissues was statistically higher when compared to normal tissues. However, the concentrations of this parameter in the blood serum were significantly lower in the CRC group. These discrepancies were explained by the authors by the different origin of CXCL5 (leukocytes, epithelial and endothelial cells) and by immunologic imbalance in the case of CRC patients. Perhaps an additional factor influencing these differences was the degree of neoplastic lesions advancement, as in the studies conducted by Dimberg et al.^18^ most of the patients were classified as Duke's A and B grades, and in our results, CXCL5 showed the highest concentration in case of patients with distant metastases (TNM stage IV).

As there are only a few studies concerning the concentration of CXCL5 in the course of human colorectal cancer, we decided to focus alsoon the studies regarding tissue and gene expression, and studies on CXCL5 in other species. All the studies indicate the high usefulness of this parameter in the course of CRC. For example, in the work of Baier et al.^19^ it was proved that the concentration of this parameter in cancer tissue is significantly higher compared to normal tissue. Similar results were also obtained by other researchers such as Hu et al.^20^, Yu et al.^21^, Meng et al.^22^, Rubie et al.^23^ and Zhang et al.^24^. In the case of the two last mentioned studies, the authors additionally performed an analysis of gene expression (qRT-PCR), which, along with similar results obtained in the work of Novillo et al.^25^, confirmed the increase of CXCL5 in the course of CRC. Also in TCM (tumor conditioned media), similar results were obtained^26^. All the above-mentioned studies conclude unequivocally that CXCL5 is a very important factor in the progression of colorectal cancer and may be a useful prognostic factor. We have also found studies pointing to elevated CXCL5 expression in murine models^27^, also those fed with a high-fat diet^28^. This may indicate a relationship of this parameter with obesity, which indicates the importance of our research, as obesity was one of the excluding parameters. However, in the absence of statistical significance obtained in the studies by Dimberg et al.^18^, it would be worth analyzing these reports.

Our attention was also drawn to the work of Zhao et al.^29^ in which the authors conducted research on CXCL5 and its involvement in the metastasis process. The authors confirmed our reports about the high involvement of this chemokine in these processes and proved that CXCL5 is produced mainly by cancer epithelial cells to induce angiogenesis. Interestingly, the authors demonstrated that overexpression of this chemokine enhanced the migration and invasion of colorectal cancer cells by inducing the epithelial-mesenchymal transition (EMT) through activation of the ERK/Elk-1/Snail pathway and the AKT/GSK3β/β-catenin pathway in a CXCR2-dependent manner. They concluded that CXCL5 may serve as a promoter of colorectal cancer metastasis and a predictor of poor clinical outcomes in colorectal cancer patients and inhibition of the CXCL5/CXCR2 signaling pathway may be a promising target for CRC therapy. Also, other authors revealed, that CXCL5 expression is required for angiogenesis, which is the first step to distant metastasis^30^.

In the case of CXCL14, we have observed a statistically lower concentration of this parameter in the serum of colorectal cancer patients when compared to healthy volunteers. Unfortunately, we did not found any similar works which could confirm or deny our studies. In the case of CXCL14 gene or tissue expression, there were few studies which partially confirmed our investigations. For example, in paper of Lin et al.^31^ using both PCR and IHC methods, the authors showed significantly lower expression of CXCL14 when compared to normal mucosa. Similarly, paper of Cao et al.^32^ revealed methylation and silencing of CXCL14 in 5 different CRC cell lines. These authors concluded, that restoration of CXCL14 expression suppressed CRC proliferation, inhibited its migration, invasion, and epithelial-to-mesenchymal transition. These findings seem extremely important as this information could provide a new target for the treatment of colorectal cancer. Moreover, it can be assumed that, potentially, the high concentration of this parameter in the course of CRC may be a positive prognostic factor for CRC patients. However, some other researchers^33^ revealed, that CXCL14 mRNA expression was higher in the case of CRC tissues. Also, research conducted by Zeng et al.^34^ proved that CXCL14 is involved in the proliferation and migration of ROS-induced CRC cells, as the expression level of CXCL14 was elevated in CRC cell lines treated with H2O2. The observed discrepancies significantly drew our attention. However, they were also noticed by other researchers^35^. The mechanisms that control the CXCL14 functions are hypothetically defined by the cell and tissue types that synthesize CXCL14 and respond to CXCL14 concentration, as well as by other proteins that co-operate with CXCL14. Mostly, CXCL14 produced by epithelial cells has been shown to overwhelm the tumor cells growth. On the other hand, CXCL14 produced from cancer-associated fibroblasts stimulates tumor growth and metastasis. While CXCL14-mediated tumor suppression and altered expression correlates with better patient survival in cancer such as head and neck, colorectal, and liver, CXCL14-mediated tumor promotion mostly occurs in tumors of the breast and pancreas. Accordingly, further studies concerning CXCL14 are needed.

Taking into account the concentration of CXCL16, we showed no statistically significant differences. However, the concentrations of this parameter in the study group were higher than in the control group. Nevertheless, the work of different research groups^33,36–41^ has shown that the concentration and expression (both tissue and mRNA) of CXCL16 in the group of patients with colorectal cancer is higher compared to the control group, the same as in investigations with use of cell lines. In addition, Chen et al.^41^ revealed that CXCL16-positive tissue expression was significantly related to tumor size, its differentiation and distant metastasis. Comparing these results to the results of our analysis using the Mann–Whitney U test, it can be assumed that we showed a similar trend, as this parameter seems to be useful in differentiation between CRC stages and demonstrated statistical significance in stage IV (distant metastases) of CRC compared to healthy controls. Perhaps, after enlarging the study group, it would be possible to prove statistical significance also in our research. Interestingly, some authors indicate that CXCL16 levels may promote tumor angiogenesis after minimally invasive colorectal resection^42^ and demonstrate the inhibitory effect on liver metastasis^43^.

The only investigators who assessed the diagnostic usefulness of CXCL5 in the blood serum of CRC patients were Yilidirim et al.^17^. They showed that the AUC for CXCL5 was 0.671, while SE, SP, PPV and NPV were 57%, 67%, 41.94% and 75%, respectively. The SE and PPV obtained by these researchers were lower than ours, while the SP, NPV and AUC were higher. These differences may result from the calculated cut-off point in both studies, which is related to the differences between the test and control groups. Interestingly, the authors, as in the case of our results, obtained slightly lower AUC for the tested parameter than the AUC of the routinely used marker, which is CEA. Regrettably, we have not found any further studies that would emphasize demonstrating the dependency and statistical significance based on the division of the tested group into advancement stages. Consequently, we believe that this work is inventive in this matter, which significantly raises its value. A more precise demonstration of the associations between the control and study group may meaningfully affect the understanding of alterations in the development of CRC.

Remarkably, our results presented significant alterations between the CXCL5 concentration in stage IV/III + IV of CRC and healthy controls, and significant differences between all TNM stages, which may indicate its contribution to the development of tumor progression and distant metastasis. What is more, CXCL14 showed statistical differences only between the control group and stage I/I + II of CRC, which, in connection with the previously acquired information about the decreasing concentration of this parameter in the course of CRC, may indicate an attempt of healthy cells to equalize CXCL14 concentration in order to prevent tumor progression, or on the contrary, about the collapse of the organism’s antitumoral action, depending on the adopted functions performed by CXCL14. Due to the fact that these are one of the first reports on these dependencies, it is desirable to confirm them in further analysis.

We also determined the correlation coefficients between the studied parameters, which confirmed that the concentration of CXCL5, CXCL16 and both tumor markers are closely related to the severity of CRC. Moreover, both CXCL5 and CXCL16 positively correlated with CEA, and CXCL16 additionally with CRP concentration. It may be related to the ongoing inflammation during the cancer progression. Unfortunately, in the available literature, we did not find any papers that could confirm or contradict the results obtained, which proves the innovation of our work.

In addition, we have proved, that simultaneous CXCL14 and CRP determinations might be more useful in CRC diagnosis than commonly used tumor marker – CEA, and CRP combination. As in the case of the above-mentioned analyzes, there are no studies available that would assess the parameters tested in a similar way.

Despite many interesting aspects, our work has some limitations. Performing additional determinations in the tissue material using alternative methods such as immunohistochemistry (IHC) on the paraffin block material and using the semi-quantitative method to compare their expression would certainly allow to confirm that the differences in chemokine levels are in fact due to the activity of tumor cells. That is why the research described in this work can be considered preliminary. Therefore, after obtaining promising results in this work, we plan to continue our research using tissue material.

## Conclusions

The present study, according to our knowledge, is the first to compare the diagnostic utility of CXCL5, CXCL14 and CXCL16 with the well-established colorectal cancer tumor markers such as CEA and CA 19–9, and CRP (the marker of inflammation), not only in the whole group of colorectal cancer patients, but also after division to TNM stages I-IV. The obtained results suggest that CXCL5 and CXCL16 may play a role in detection of distant metastases and differentiation between TNM stages, as well as combination of CXCL14 and CRP as potential CRC biomarkers. These proteins could also prove useful in detecting disease recurrence after treatment. However, further studies concerning the concentrations of selected CXC chemokines in the course of CRC are necessary to confirm and clarify their diagnostic usefulness and clinical application as potential biomarkers of CRC development.

## Data Availability

The data presented in this study are available on request from the corresponding author. Key data are stated in the text.
